# Potentiating the Activity of Nisin against *Escherichia coli*

**DOI:** 10.3389/fcell.2016.00007

**Published:** 2016-02-08

**Authors:** Liang Zhou, Auke J. van Heel, Manuel Montalban-Lopez, Oscar P. Kuipers

**Affiliations:** Department of Molecular Genetics, Groningen Biomolecular Sciences and Biotechnology Institute, University of GroningenGroningen, Netherlands

**Keywords:** lantibiotic, nisin, fusion, Gram-negative, outer membrane

## Abstract

Lantibiotics are antimicrobial (methyl)lanthionine-containing peptides produced by various Gram-positive bacteria. The model lantibiotic, nisin, binds lipid II in the cell membrane. Additionally, after binding it can insert into the membrane creating a pore. Nisin can efficiently inhibit the growth of Gram-positive bacteria and resistance is rarely observed. However, the activity of lantibiotics is at least 100-fold lower against certain Gram-negative bacteria. This is caused by the fact that Gram-negative bacteria have an outer membrane that hinders the peptides to reach lipid II, which is located in the inner membrane. Improving the activity of lantibiotics against Gram-negative bacteria could be achieved if the outer membrane traversing efficiency is increased. Here, several anti-Gram-negative peptides (e.g., apidaecin 1b, oncocin), or parts thereof, were fused to the C-terminus of either a truncated version of nisin containing the first three/five rings or full length nisin. The activities of these fusion peptides were tested against Gram-negative pathogens. Our results showed that when an eight amino acids (PRPPHPRL) tail from apidaecin 1b was attached to nisin, the activity of nisin against *Escherichia coli* CECT101 was increased more than two times. This research presents a new and promising method to increase the anti-Gram-negative activity of lantibiotics.

## Introduction

Lantibiotics are ribosomally synthesized and post-translationally modified peptides. After modification, they consist of one or more (methyl)lanthionine rings, dehydroalanines, or dehydrobutyrines. Additionally, some lantibiotics display additional modifications (Willey and van der Donk, 2007). Most lantibiotics inhibit the growth of Gram-positive bacteria using lipid II as a target molecule (Bauer and Dicks, 2005). Lipid II plays an essential role in cell-wall synthesis. Diverse lantibiotics bind to the pyrophosphate group in lipid II, and subsequently form pores in the membrane, which is fatal for the bacteria (Breukink et al., 1999; Hasper et al., 2006). Specific resistance to lantibiotics is therefore rarely found (Breukink and de Kruijff, 2006; Draper et al., 2015). However, Gram-negative bacteria have an outer membrane, which is composed of a phospholipid layer (inside) and an outside layer of lipopolysaccharide (LPS) which contains lipid A and polysaccharide chains (Erridge et al., 2002). The LPS is highly negative-charged and the core oligosaccharide region is ordered by divalent cations (mainly Ca^2+^ and Mg^2+^) (Clifton et al., 2015), which can hamper lantibiotics from reaching lipid II in the inner membrane. Thus, enhancing the activity of lantibiotics against Gram-negative pathogens first requires improving the outer membrane penetration capability.

**Figure 1:**
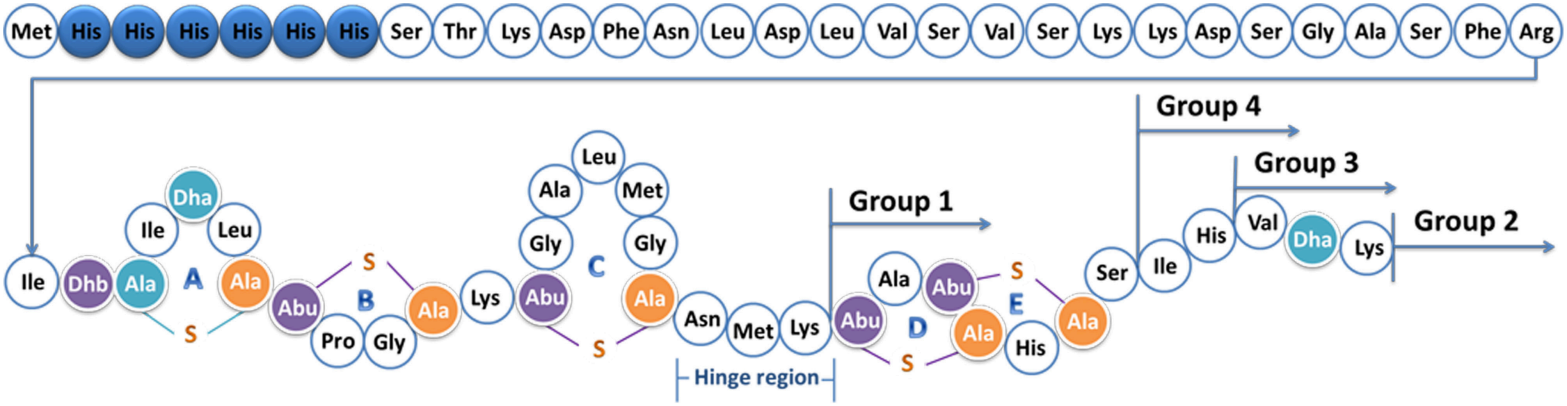
**Structure of prenisin with his-tag**. The six histidine residues are labeled in blue. Dha, dehydroalanine; Dhb, dehydrobutyrine; Ala-S-Ala, lanthionine; Abu-S-Ala, β-methyllanthionine. The rings ABCDE and the hinge region (Asn- Met-Lys) are marked; the positions of the tails are indicated. The molecular weight is 6640.7 Da.

Nisin (Figure 1) produced by *Lactococcus lactis* is the first identified lantibiotic (Lubelski et al., 2008). The structural gene of nisin encodes a 57 amino acids prepeptide. The first 23 amino acids form the leader part and the last 34 residues constitute the core peptide. The leader peptide guides the core peptide through the modification and transport system, and keeps nisin inactive (Plat et al., 2013). Firstly, NisB dehydrates the serines and threonines to form dehydroalanines (Dha) or dehydrobutyrines (Dhb) (Ortega et al., 2015). Then NisC couples the cysteine to the Dha or Dhb by a sulfhydryl addition reaction (Kuipers et al., 1993; Koponen et al., 2002). The modified peptide is transported to the outside of the cell by NisT (Kuipers et al., 2004). NisP is a protease specifically cutting off the leader peptide liberating active nisin (van der Meer et al., 1993). After modification, the peptide contains five (methyl)lanthionine rings, two Dha residues, and one Dhb. NisB and NisC have a relaxed substrate specificity, and when the core peptide is replaced by other sequences, the modifications can still be performed in most cases (Kluskens et al., 2005; Rink et al., 2007a; Majchrzykiewicz et al., 2010).

Nisin can efficiently inhibit the growth of Gram-positive bacteria, with a minimal inhibitory concentration in the nanomolar range. The rings A and B of nisin can bind to the pyrophosphate of lipid II on the membrane of Gram-positive bacteria by forming a pyrophosphate cage (Hsu et al., 2004) and consequently inhibit cell wall synthesis. The N-terminal part is essential for the activity of nisin, and if the C-terminal part is deleted, moderate activity is still observed (Rink et al., 2007b). After binding, the C-terminal part of nisin can translocate across the membrane and form pores by assembling a pore complex in a stoichiometry of 8 nisin and 4 lipid II molecules (Breukink and de Kruijff, 2006).

Nisin displays much lower activity against most Gram-negative bacteria, because the outer membrane can prevent the peptide to reach the periplasm and to exert activity binding lipid II in the inner membrane. When the outer membrane is destabilized using ethylenediaminetetraacetic acid (EDTA) or pyrophosphate, nisin can inhibit the Gram-negative bacteria more efficiently (Boziaris and Adams, 1999; Helander and Mattila-Sandholm, 2000). This indicates that passing the outer membrane is crucial for the activity of nisin against Gram-negative bacteria.

Notably, there are some antimicrobial peptides (AMPs) which can efficiently inhibit the growth of Gram-negative bacteria, such as apidaecin 1b, oncocin, or EC5 (Table 1). These peptides are normally positively charged, some are proline-rich and they all can efficiently traverse the outer membrane. In this paper we aim to design and produce hybrid molecules that combine the lipid II binding capacity of nisin with the ability of these eukaryotic peptides to cross the outer membrane of Gram-negative bacteria. Thus, the anti-Gram negative peptides or the membrane-translocating part of them were attached to nisin or to the N-terminal part of nisin, and the activities of the fusions against Gram-negative bacteria were tested. One fusion was found to have higher activity than nisin, which indicates the potential of this approach. This is the first attempt to potentiate the activity of nisin against Gram-negative microorganisms by adding a tail that could facilitate traversing the outer membrane.

**Table 1:** **List of selected peptides with anti-Gram-negative bacteria activity**.

**Name**	**Sequence^a^**	**MIC(μM)**	**References**
Apidaecin 1b	GNNRPVYIPQPRPPHPRL	0.5^b^	Berthold et al., 2013
Api 88	Gu-ONNRPVYIPRPRPPHPRL-NH_2_	0.2^b^	Czihal et al., 2012
Oncocin	VDKPPYLPRPRPPRRIYNR-NH_2_	1.7^b^	Knappe et al., 2010
Drosocin	GKPRPYSPRPTSHPRPIRV	25^c^	Bikker et al., 2006
EC5	RLLFRKIRRLKR	4.8^d^	Sainath Rao et al., 2013
Bac8c	RIWVIWRR-NH_2_	1.7^e^	Hilpert et al., 2005
R-BP100	KKLFKKILKYL-NH_2_	0.9 ± 0.4^f^	Torcato et al., 2013
RW-BP100	RRLFRRILRWL-NH_2_	0.5 ± 0.2^f^	Torcato et al., 2013
ADP2	GIGKHVGKALKGLKGLLKGLGEC-NH_2_	1^f^	Iliæ et al., 2013
8R^g^	RRRRRRRR	ND	Wender et al., 2000

a Gu denotes N,N,N',N'-tetramethylguanidine, and O denotes L-ornithine.The indicator strains used for minimum inhibitory concentration (MIC) tests were

b E. coli BL21 AI;

c E. coli O157:H7;

d E. coli ATCC 700928;

e E. coli UB1005;

f *E. coli ATCC 25922*.

g *Only has membrane penetrating activity. ND, not determined*.

## Materials and methods

### Bacterial strains and growth conditions

The bacterial strains used in this study are listed in Table 2. *L. lactis* strains were cultured in M17 broth supplemented with 0.5% (w/v) glucose (GM17) for genetic manipulation or in minimal expression medium (MEM) for protein expression at 30°C (Rink et al., 2005). *E. coli* CECT101 was grown in Luria-Bertani broth aerated by shaking (200 rpm) at 37°C.

**Table 2:** **Strains and plasmids used in this study**.

**Strains or plasmids**	**Characteristics**	**References**
**STRAINS**		
*Lactococcus lactis* NZ9000	*nisRK*	Kuipers et al., 1997
*L. lactis* PA1001	Derivative of NZ9000, with *acmA* and *htrA* deleted	Bosma et al., 2006
**PLASMIDS**		
pIL3EryBTC	*nisBTC*, encoding nisin modification machinery, EryR^a^	van Heel et al., 2013
pNZ8048	Nisin inducible promoter in shuttle vector	de Ruyter et al., 1996
pNZnisA	*nisA*, encoding nisin, CmR^b^, inserted in pNZ8048	van Heel et al., 2013
pNZnisA leader6H	*nisA*, encoding nisin, with 6 histidine residues inserted behind the first methionine	This study
pNZnisA GNT16	*nisA*, encoding nisin, with 6 histidine residues inserted behind the first methionine and tail PRPPHPRL fused to the C-terminus	This study
pNZnisA GNTs	*nisA*, encoding nisin or part of nisin, with 6 histidine residues inserted behind the first methionine and tails listed in Table 3	This study
pNZnisP8H	*nisP*, encoding a NisP mutant, with 8 histidines, CmR^b^	Unpublished data
**INDICATOR STRAINS**		
*L. lactis* MG1363	Nisin sensitive indicator	Gasson, 1983
*Escherichia coli* CECT101	Gram-negative indicator	CECT

a *EryR, erythromycin resistance*.

b *CmR, chloramphenicol resistance*.

### Molecular cloning

A 6 his-tag was added to the leader part of nisin by PCR based on the plasmid pNZnisA as described previously (Zhou et al., 2015). Standard molecular cloning was performed according to Sambrook and Russell (2001). The tails were added to nisin by designing primers containing the sequences of the tail and a selected part of nisin with SacI and HindIII at either end. The primers were annealed according to the protocol on the website of Sigma-Aldrich (^1^Protocol for Annealing Oligonucleotides). The annealed double strand DNAs were ligated to the pNZnisA leader6H vector cut by SacI and HindIII. Competent cells were prepared and transformed as described previously (Holo and Nes, 1995).

### Protein expression, TCA precipitation, and tricine SDS-PAGE

The expression of the peptides was conducted using *L. lactis* NZ9000 or *L. lactis* PA1001 containing the plasmids pIL3EryBTC and pNZnisA leader6H harboring the nisin and anti-Gram negative tail fusion. The culture and expression methods were the same as previously described (Zhou et al., 2015). Cells were cultured at 30°C first in GM17 medium with 4 μg/ml chloramphenicol and 4 μg/ml erythromycin until OD (600 nm) reached 0.7, then centrifuged and resuspended in the same amount of MEM medium with 0.5% (w/v) glucose, 3 μg/ml chloramphenicol, 3 μg/ml erythromycin, and 2 nM nisin to induce the expression of the peptides. After 3 h induction, the supernatant was harvested. The supernatant of a small volume of fermentation (< 10 ml) was concentrated by trichloroacetic acid (TCA) precipitation (Sambrook and Russell, 2001) and the concentrated peptides were loaded on a 16% Tricine SDS-PAGE gel (Schägger, 2006). NisP was expressed to cleave off the leader part of the peptides. The strain NZ9000 (pNZnisP8H) was cultured and harvested in the same way as above, but here only chloramphenicol was added.

### Purification, characterization, and quantification

For large scale purification, 2 L supernatant containing the mature prepeptide and 100 ml supernatant containing NisP were filtered (0.2 μm membrane, Millipore), mixed together and incubated at 30°C for 1 h to cut off the leader peptide. After incubation, the active peptides were first purified by cation-ion exchange chromatography (van Heel et al., 2013). Then, the eluate was loaded on a C18 (Spherical C18, Sigma-Aldrich) column. The peptides were eluted with 30–40% buffer B (Buffer A, miliQ with 0.1% trifluoroacetic acid (TFA); Buffer B, isopropanol: acetonitrile (2:1) with 0.1% TFA). The elutions from the C18 column were freeze dried. The freeze dried peptides were further purified by HPLC (Agilent 1260 Infinity LC) equipped with a semi-preparative C12 column (Phenomenex 250 × 10 mm, 4 μm, Proteo 90Å) as described previously (Zhou et al., 2015). The fractions were collected, tested for activity against *L. lactis* and analyzed by MALDI-TOF as described previously (van Heel et al., 2013). The active, fully dehydrated, and pure fractions were freeze dried and quantified with HPLC as described previously (Zhou et al., 2015).

### Determination of the minimum inhibitory concentration (MIC)

The indicator strains were first cultured until OD (600 nm) reached 0.5. When testing the MIC value, the culture was diluted 1000 times with the appropriate medium. All the tests were performed with a temperature controlled plate reader (Tecan infinite F200, Tecan Group AG) in a 96-well plate (Greiner Bio-one). The peptides were first diluted in gradient with medium and then mixed with diluted indicator strains. The final concentration of the peptides ranged from 0.00625 to 0.8 μM against *L. lactis* MG1363 and from 0.25 to 32 μM against *E. coli* CECT101 in 100 μl volume. The plate was incubated at 30°C or 37°C for 18 h depending on the indicator strain being *L. lactis* or *E. coli*, respectively. OD (600 nm) was checked every 30 min. For *E. coli*, 2 min of shaking was performed before every check. When testing the activity of nisin against *E. coli* in the presence of EDTA, an EDTA solution was prepared and added to a final concentration of 50, 110, or 250 μM. The minimal concentration of peptide causing no observed growth of indicator strains was considered as the MIC value.

## Results

### The anti-gram negative tails are attached to different parts of nisin

To increase the outer membrane penetration capability of nisin, 10 different anti-Gram-negative peptides were combined with nisin in four different ways (Figure 1, Table 3). More specifically, nisin binds to the lipid II molecule with its first two rings, a process that consequently inhibits the synthesis of the cell wall. Moreover, a mutant of nisin where the amino acids 23–34 were deleted (i.e., rings DE and the C-terminal linear part of nisin) still retains a modest antimicrobial activity (Rink et al., 2007b). We hypothesized that by combining the ABC rings of nisin and an anti-Gram-negative peptide tail, the fusion could gain the potential to traverse the outer-membrane, while maintaining the lipid II binding activity, thereby inhibiting growth. Based on this idea, 13 different peptides were designed (Table 3 Group 1). In the cases of apidaecin 1b and oncocin, different regions of the peptides were linked to the first three rings of nisin. Additionally, the PRPPHPRL tail of apidaecin 1b was added either alone (GNT16-3rings) or as a duplicated motif (GNT5).

**Table 3:** **Sequences of nisin and anti-Gram-negative tail fusions**.

	**Peptides**	**Sequence**
Group 1	Architecture	Ring ABC + hinge region + tail
	GNT1	Ring ABC +NMKVYIPRPRPPHPR
	GNT1+L	Ring ABC +NMKVYIPRPRPPHPRL
	GNT4	Ring ABC + NMKGNNRPVYIPRPRPPHPRL
	GNT5	Ring ABC + NMKPRPPHPRLNMKPRPPHPRL
	GNT16− 3 rings	Ring ABC + NMKPRPPHPRL
	GNT6	Ring ABC + NMKPPYLPRPRPPRRIYNR
	GNT7	Ring ABC + NMKPRPRPPRRIYNR
	GNT8	Ring ABC + NGKPRPYSPRPTSHPRPIRV
	GNT2	Ring ABC + NMRLLFRKIRRLKR
	GNT10− 3 rings	Ring ABC + NMRIWVIWRR
	GNT11− 3 rings	Ring ABC + NMKLFKKILKYL
	GNT12− 3 rings	Ring ABC + NMRRLFRRILRWL
	GNT15− 3 rings	Ring ABC + NMGKHVGKALKGLKGLLK
Group 2	Architecture	Nisin + tail
	GNT16	Nisin + PRPPHPRL
	GNT17	Nisin + PRPRPPRRIYNRN
	GNT10	Ring ABCDE + SIHVSRIWVIWRR
	GNT11	Nisin + LFKKILKYL
	GNT12	Ring ABCDE + SIHVSRRLFRRILRWL
	GNT15	Nisin + GKHVGKALKGLKGLLK
	Nisin + 8 R	Nisin + RRRRRRRR
Group 3	Architecture	Nisin △VSK+ tail
	GNT16ΔVSK	Nisin △VSK + PRPPHPRL
	GNT16ΔIHVS	Nisin △IHVS + PRPPHPRL
	GNT10ΔVSK	Nisin △VSK + RIWVIWRR
	GNT12ΔVSK	Nisin △VSK + RRLFRRILRWL
	GNT15ΔVSK	Nisin △VSK + GKHVGKALKGLKGLLK
Group 4	Architecture	Ring ABCDE + SG + tail
	GNT2SG	Ring ABCDE + SG + RLLFRKIRRLKR
	GNT3SG	Ring ABCDE + SG + RIWVIWRR
	GNT12SG	Ring ABCDE + SG + RRLFRRILRWL
	GNT15SG	Ring ABCDE + SG + GKHVGKALKGLKGLLK
	GNT16SG	Ring ABCDE + SG + PRPPHPRL
	GNT17SG	Ring ABCDE + SG + PRPRPPRRIYNRN
	GNT3	Ring ABCDE+RIWVIWRR

*The additional anti-Gram-negative tail is underlined*.

The full length nisin can additionally form pores in the cytoplasmic membrane after binding to lipid II, which increases its potency as compared to lipid II sequestering by the first three rings (Breukink and de Kruijff, 2006). Therefore, in this research the anti-Gram negative tails were also fused behind full length nisin (Table 3 Group 2). In those cases where the anti-Gram-negative tail was starting with a positively charged amino acid, Lys34 of nisin was deleted (i.e., GNT10 and GNT12).

As some of the Group 2 peptides (e.g., GNT15) tend to be truncated behind Val32 (data not shown), part of the C-terminal sequence of nisin was deleted in some of the variants (Table 3 Group 3). Following a similar reasoning, the C-terminus of nisin behind ring E was deleted and instead a Ser-Gly linker was added as a flexible linker in front of the tail (Table 3 Group 4). An exception in Group 4 is GNT3, because in this case the anti-Gram-negative tail is directly pasted behind ring E with no linker in between.

### The fusions show strongly varying production levels

**Figure 2:**
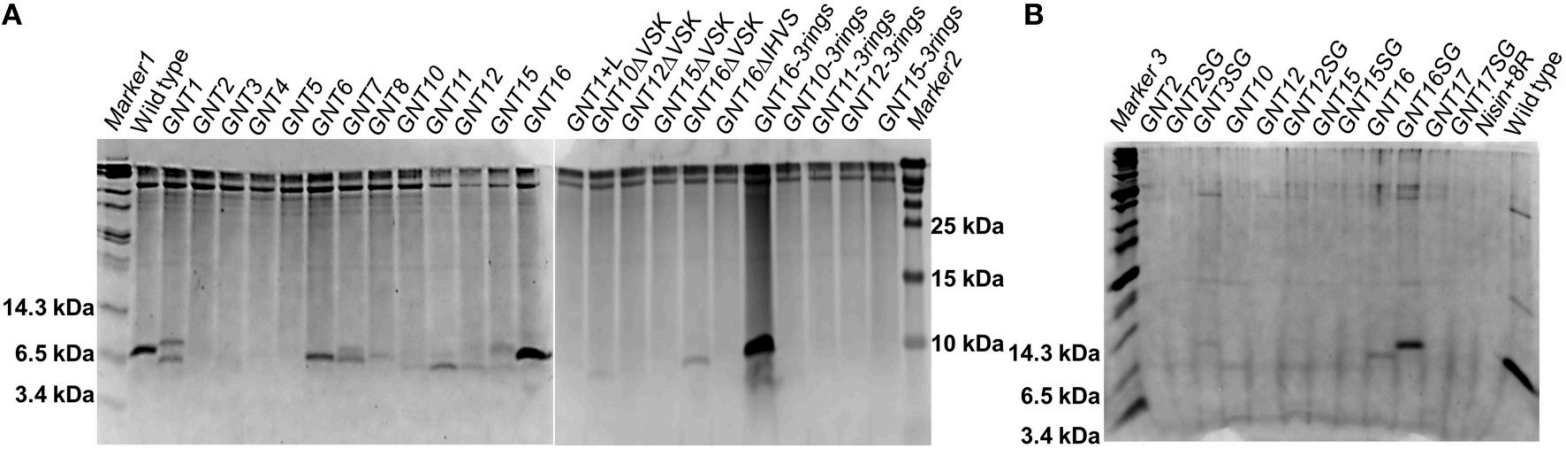
**Coomassie-blue stained tricine SDS-PAGE gel**. The fusion peptides were produced with NZ9000 **(A)** or with PA1001 **(B)**. Each well contains TCA precipitated peptides from 600 μl supernatant. All the samples are prepeptides with the leader and his tag part still attached. The experiment was performed several times showing similar results.

The nisin and anti-Gram-negative tail fusions were produced by the nisin inducible production system previously described, which consists of NZ9000(pIL3EryBTC, pNZ8048-nisin derivative) (Rink et al., 2005; van Heel et al., 2013). The production levels were monitored by TCA precipitations of the supernatants analyzed by tricine SDS-PAGE (Figure 2A). The production levels of the fusion peptides vary greatly, mainly depending on the types of the tail, e.g., the variants containing the tail from apidaecin 1b (GNT1, GNT16 and GNT16-3rings), oncocin (GNT6 and GNT7) and drosocin (GNT8) showed good production levels. The fusions containing other kinds of tails showed low production levels. Additionally, the MALDI-TOF analysis showed that some variants were partly degraded (data not shown). Furthermore, with the same kind of tail, the design rules also affect the production level, e.g., GNT16-3rings showed generally higher expression levels than GNT1 and GNT16.

To reduce the amount of extracellular proteases and obtain more intact peptides, the deletion strain PA1001 (Δ*acmAΔhtrA*) (Bosma et al., 2006) was tested to express some of the fusions (Figure 2B). In this system, wild type nisin showed almost the same production level as in the NZ9000 system, but the amount of contaminant proteins was much less. In this case, the GNT3SG (containing Bac8c) was expressed in high amounts. The mutants GNT16 and GNT16SG also showed good production and the production level of GNT16SG was even higher.

### The nisin and anti-Gram negative tail fusions were characterized by MS

The fusions with relatively higher production levels (GNT1, GNT6, GNT7, GNT8, GNT16, GNT16 -3 rings, and GNT16SG) were further purified and characterized by MS after leader peptide cleavage. After purification, the variants GNT1, GNT16, GNT16 -3rings, and GNT16SG showed almost pure peaks, while the GNT6, GNT7, and GNT8 contained degradation products (Supplementary Figure 1). The mass of the degraded peptides (Supplementary Table 1) indicates that the peptides GNT6, GNT7, and GNT8 tend to be degraded at the C-terminus, with the C-terminal R, NR, YNR, IYNR, or IRV deleted.

The dehydration extent of the intact peptides was analyzed, and the results (Table 4) showed that the GNT1, GNT6, GNT7, GNT16, GNT16 -3 rings, and GNT16SG were fully dehydrated. The GNT8 fusion contains 2 serines and 1 threonine in the tail, and the full peptide was dehydrated seven times, thus five times in the nisin part and two in the tail.

**Table 4:** **Mass of nisin and nisin with anti-Gram-negative tail fusions**.

**Peptides**	**Number of dehydrations**	**Predicted mass (Da)**	**Observed mass (Da)**
Nisin	8	3355.1	3353.5
GNT1	5	3607.5	3606.7
GNT6	5	4171.1	4169.3
GNT7	5	3700.6	3702.4
GNT8	7	4025.9	4026.5
GNT16	8	4306.3	4304.3
GNT16-3rings	5	3091.9	3092.8
GNT16SG	7	3816.7	3818.9

### Effect of EDTA on the activity of nisin against *E. coli*

Nisin normally shows low activity against Gram-negative organisms. In this research, we determined a MIC value of nisin of 16 μM against *E. coli* CECT101 (Table 5). As described before, nisin can inhibit the growth of Gram-negative bacteria when a sufficient amount of EDTA(>100 μM) was added (Helander and Mattila-Sandholm, 2000). In this research, different concentrations of EDTA were added together with nisin. The results show that as the concentration of EDTA went up, less nisin was needed to exhibit full inhibition.

**Table 5:** **Minimum inhibition concentration (MIC) of nisin against *E.coli* CECT101 in the presence of different concentration of EDTA**.

**Concentration of EDTA (μM)**	**MIC value of nisin against *E. coli* CECT101(μM)**
0	16
25	16
110	8
250	4

### The fusions displayed lower net activity than nisin against *L. lactis*

Nisin inhibits the growth of *L. lactis* MG1363 at the nanomolar range (6 nM Table 6). However, when equipped with a tail, the MIC values against *L. lactis* increased and ranged between 200 nM (GNT16 and GNT7) and 1000 nM (GNT1 and GNT16-3rings). The activity of GNT16SG against *L. lactis* was two times lower than GNT16, but 2.5 times higher than GNT16-3rings.

**Table 6:** **Activities of nisin and nisin-tail fusions**.

**Peptides**	**MIC value^a^ (μM)**	
	***E. coli* CECT101**	***L. lactis* MG1363**
Nisin	16	0.006
GNT1	>16	1
GNT6^*^	>16	ND
GNT7^*^	>16	0.2
GNT8^*^	>16	ND
GNT16	8	0.2
GNT16-3rings	>32	1
GNT16SG	>32	0.4

a *The experiments were repeated at least two times*.

* *These peptides were mixed with partial C-terminal degradation products. ND, not determined*.

### Activity of the fusions against Gram-negative bacteria

**Figure 3:**
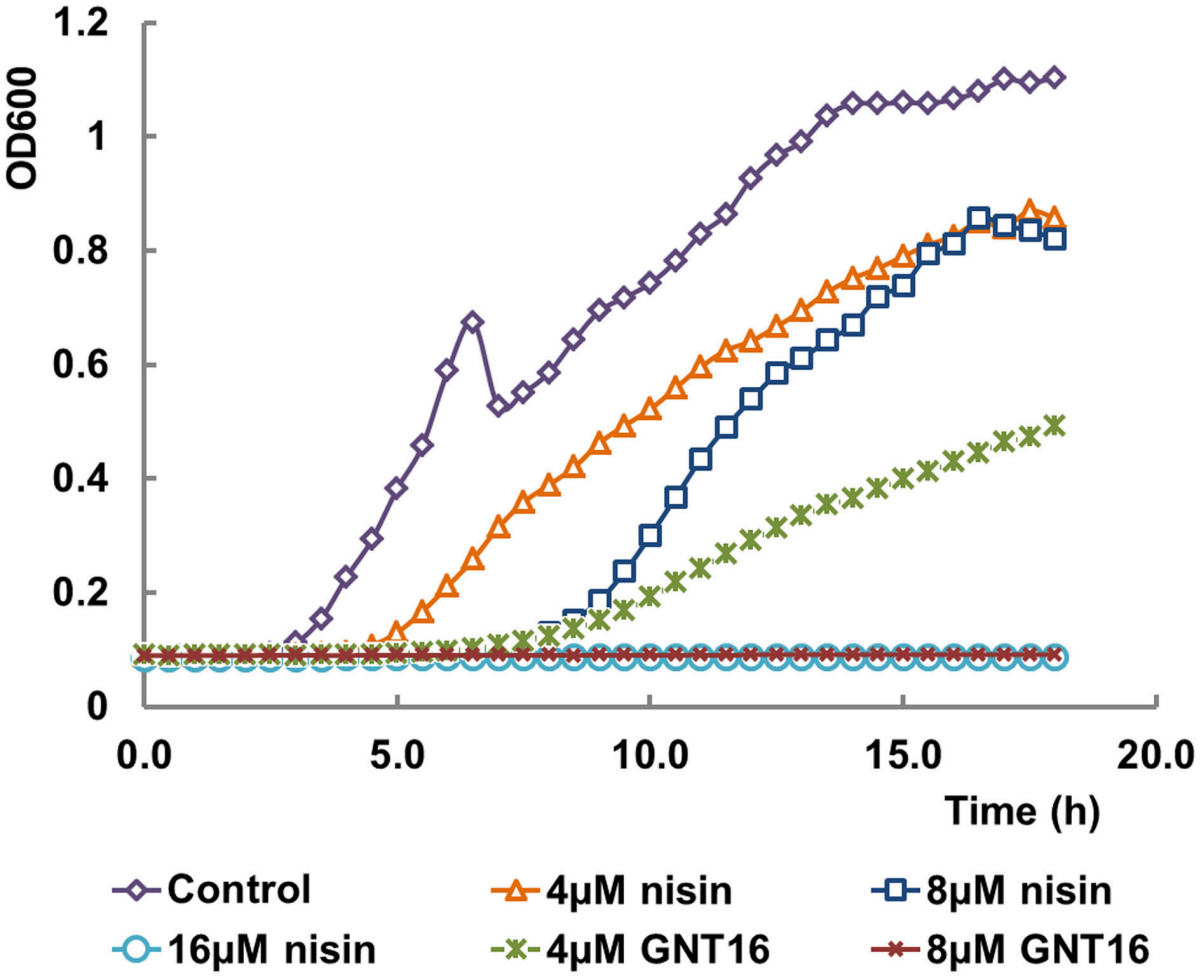
**Inhibitory effects of nisin and GNT16 against *E. coli* CECT101**. Either 16 μM nisin or 8 μM GNT16 could fully inhibit the growth of bacteria within 18 h. The experiment was repeated three times.

Table 6 shows the activities of nisin and the fusions against *E. coli* CECT101. The peptides GNT1, GNT6, GNT7, GNT8, GNT16 -3rings, and GNT16SG showed lower net activity than nisin against *E. coli*, while GNT16 displayed two times higher activity than nisin (Table 6). In addition, the mutants GNT6, GNT7, and GNT8 were a mix of intact and partly degraded peptide (Supplementary Figure 1). Figure 3 shows that 4 μM GNT16 displayed better inhibitory activity than 8 μM nisin, which means that the activity of GNT16 was more than two times higher than that of nisin. GNT16 showed a significant improvement comparable to the concentration of nisin needed in the presence of 110 μM EDTA. Furthermore, GNT16 also showed an equal or slightly better inhibitory activity than nisin against *E. coli* CIP and *Enterobacter aerogenes* CECT684, which exhibited lower growth after prolonged incubation (Supplementary Figure 2).

## Discussion

The concomitant use of nisin with other compounds to increase the inhibition effects against Gram-negatives has been described. For instance, the combination with polymyxin E (Naghmouchi et al., 2013) or with chelating agents such as EDTA (Boziaris and Adams, 1999) gave a better inhibition. There are also genetic engineering products which have improved activity. Mutating the hinge region of nisin Z and thereby increasing the positive charge was found to make nisin slightly more active against several species of Gram-negative bacteria, such as *Shigella, Pseudomonas* and *Salmonella* but not *E. coli* (Yuan et al., 2004). In the study of Field et al. (2012), S29 of nisin was mutated into G, A, or E, which resulted for all the mutants in a two-fold increased activity against *E. coli* 0157-H7 and *Salmonella enterica* serovar Typhimurium. In our research, the outer membrane-passing capacity of nisin was aided by attaching some highly positive charged (at least four positive charges) antimicrobial peptides as a tail. These AMPs are usually rich in proline, arginine, lysine, and hydrophobic amino acids. In contrast to polymyxin E and EDTA, which disrupt the stability of the outer membrane (Naghmouchi et al., 2013; Clifton et al., 2015), the AMPs go into the cells without breaking the outer membrane. They tend to form a helical structure when interacting with the negatively charged outer membrane and at the same time transit the membrane (Torcato et al., 2013). With this kind of tail as a sort of Trojan horse, the trans-outer membrane efficiency of nisin probably could be increased.

The peptide GNT16 contains a full length nisin and a tail from apidaecin 1b. Apidaecin 1b is a kind of proline-rich AMP, which inhibits the growth of Gram-negative bacteria by translocation into the cytoplasm and binding the chaperone DnaK. Api88 is a derivative of apidaecin 1b with increased positive charges and an amidated C-terminus, which displays higher activity. The crystal structure of DnaK and Api88 showed that residues 5–11(PVYIPRP) of Api88 bind to DnaK. The tail (PRPPHPRL), which does not display DnaK binding activity, probably has a good outer membrane and inner membrane-passing capability (Czihal et al., 2012). In this research, the nisin-PRPPHPRL fusion showed increased activity compared to nisin against *E. coli*, which is probably because the tail has improved the trans-outer membrane capability of the peptide. Furthermore, the fusion GNT16 showed 32 times decreased activity against *L. lactis* MG1363, which means that attaching the tail is detrimental for the original inhibition activity of nisin probably due to the elimination of the pore forming ability of nisin (Rink et al., 2007b).

Interestingly, the part of nisin that is retained in the fusion affects the activity of the fusion peptide. The GNT16SG displayed two times lower activity than GNT16 against *L. lactis*, and more than four times lower activity against *E. coli*. The GNT16-3 rings displayed even more reduced activity than GNT16SG against both strains. This indicates that the intertwined rings and the last six amino acids of nisin are helpful for the activity. Normally, a helical conformation is formed during the AMPs passing the outer membrane. The C-terminus of nisin has a helical structure (van de Ven et al., 1991) as well, which may be helpful for the outer membrane passing capacity. Alternatively, a longer linker could be beneficial for the tail from apidaecin 1b to form a PP II helix (polyproline helical type II) (Li et al., 2006), and traverse the outer membrane.

The fusion GNT7 consists of the first three rings of nisin and a PRPRPPRRIYNR tail from oncocin. The partly purified GNT7 displayed equal activity as GNT16 against *L. lactis* and higher activity than GNT16-3rings and GNT16SG, which means that the GNT7 possibly displays relatively better pore formation activity.

We have seen that all the peptides in this research are active against *L. lactis* (data in Supplementary Figure 3). This is in accordance with the literature, which indicates that the first three rings of nisin retain activity (Rink et al., 2007b). However, as most of the peptides were expressed at very low levels or were degraded after expression, full length fusions are difficult to obtain. To overcome this problem, some *in vitro* synthesis methods (e.g., solid-phase peptide synthesis) might be employed to increase the success rate of this novel design (Montalbán-López et al., 2012). Some kinds of AMPs (e.g., Api 88, oncocin, Bac8c, R-BP100, RW-BP100, and ADP2) contain a C-terminal amide, which can increase the activity. If the fusion peptides could be amidated as well, probably the activities against Gram-negatives could be enhanced. However, to the best of our knowledge there are no *in vivo* amidation strategies available in *L. lactis*. The stability of the tails is very important for the production level of the fusions. The seven fusions obtained in this research contain tails from apidaecin 1b, oncocin, and drosocin, which indicates that proline-rich AMPs are more stable and easier to be expressed in *L. lactis*. The production level of GNT16-3rings > GNT16SG > GNT16> GNT16ΔVSK > GNT16ΔIHVS, which indicates that a shorter part of nisin (first three or five rings) can render a higher production level but also that the last five residues of nisin form a weak region.

Compared to apidaecin 1b which can inhibit the Gram-negative organisms at 0.5 μM (Berthold et al., 2013), the best fusion peptide showed still a 16-fold higher MIC value, although the experimental setup is different. To increase the activity of nisin against Gram-negative bacteria, both the inhibition activity and trans-outer membrane activity are important. When the first three rings of nisin are intact, the lipid II binding activity is stable. Retaining the pore formation activity is crucial. By further variation of the linker between the nisin part and the tail or searching for more efficient tails, higher pore formation activity and trans-outer membrane efficiency can undoubtedly be obtained. Moreover, after the fusion peptides enter the periplasm, particular periplasmic proteases might be used to liberate both compounds. In this way, both nisin and the anti-Gram-negative peptides could perform their inhibitory activity more efficiently although this point need further study. The periplasmic HtrA protease might be a good candidate to cleave the fusions (Lipinska et al., 1990). Our data combining in a single nisin derivative the lipid II binding activity of nisin with the penetrating activity of eukaryotic antimicrobial peptides indicate that a rational design can improve the activity of lantibiotics against Gram-negative bacteria.

## Author contributions

OPK contributed to the conception and design of the work, analysis, and interpretation of data, and revising the work critically for important intellectual content. LZ performed the acquisition, analysis, and interpretation of data for the work, and drafted the work. AJvH and MML participated the design of the work, analysis, and interpretation of the data, and revised the manuscript critically for important intellectual content. All the authors show final approval of the version to be published and agreed to be accountable for all aspects of the work in ensuring that questions related to the accuracy or integrity of any part of the work are appropriately investigated and resolved.

### Conflict of interest statement

The authors declare that the research was conducted in the absence of any commercial or financial relationships that could be construed as a potential conflict of interest.

## Footnotes

^1^Protocol for Annealing Oligonucleotides Sigma-Aldrich. Available online at: http://www.sigmaaldrich.com/technical-documents/protocols/biology/annealing-oligos.html (Accessed April 8, 2015).
