# Supplementary material for: Potentiating the Activity of Nisin against Escherichia coli
Source: Front Cell Dev Biol. 2016 Feb 8;4:7. doi: 10.3389/fcell.2016.00007 (PMC4745983; doi:10.3389/fcell.2016.00007)
Supplement: Supplementary file 1 [file DataSheet1.PDF]

Supplementary Material

Potentiating the activity of nisin against *Escherichia coli*

Liang Zhou, Auke J. van Heel, Manuel Montalban-Lopez, Oscar P. Kuipers*

*** Correspondence:** Oscar P. Kuipers: o.p.kuipers@rug.nl

1 Supplementary Data 1

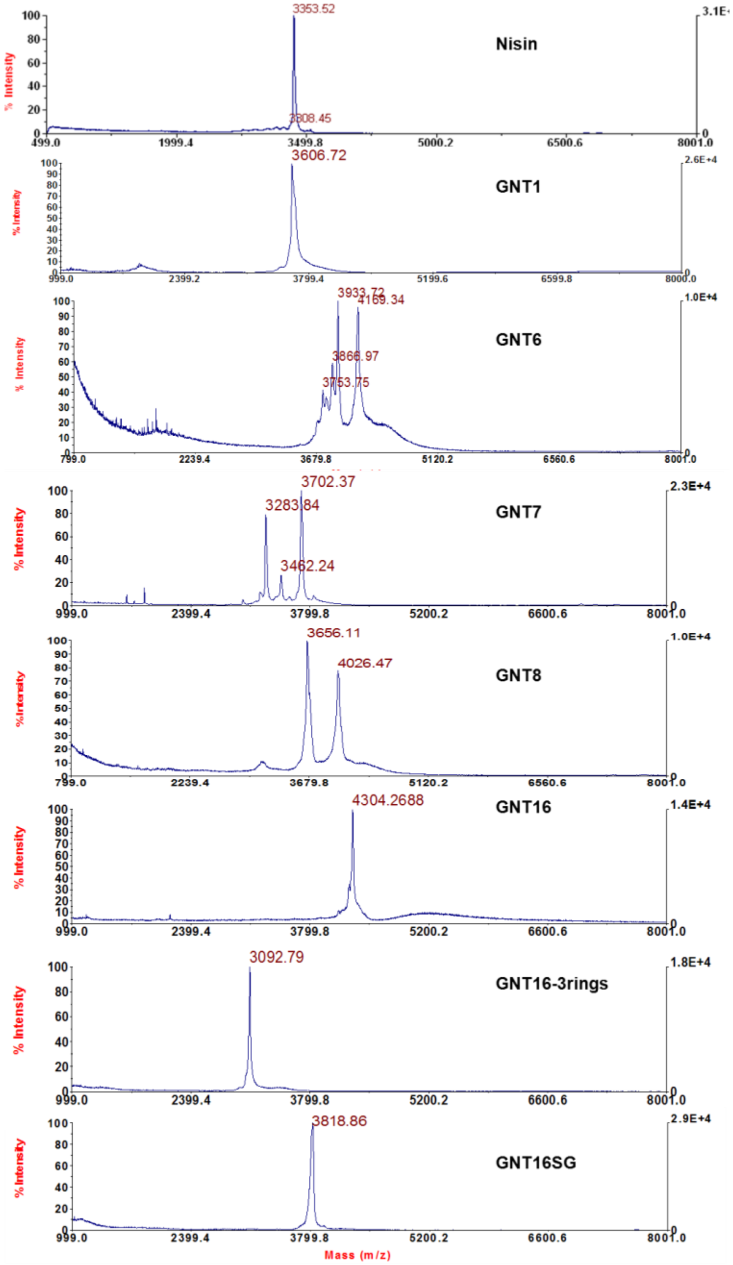

Supplementary Figure 1. Mass spectra of nisin and nisin mutants with anti-Gram-negative tails.

Supplementary Table 1. Mass of degraded nisin and anti-Gram-negative fusions detected by MALDI-TOF

**Peptides**	**Number of dehydrations**	**Predicted mass (Da)**	**Observed mass (Da)**	**Inferred degradation**
GNT6	5	4171.1	4169.3	No
	3	3936.8	3933.7	Δ NR
	3	3773.7	3774.0	Δ YNR
GNT7	5	3700.6	3702.4	No
	5	3544.4	3546.0	Δ R
	3	3466.3	3462.2	Δ NR
	2	3484.3	3484.2	Δ NR
	4	3285.1	3283.8	Δ YNR
	3	3303.1	3302.1	Δ YNR
	2	3321.1	3319.9	Δ YNR
	5	3153.9	3157.0	Δ IYNR
GNT8	7	4025.9	4026.5	No
	7	3657.4	3656.1	Δ IRV
	6	3675.4	3674.3	Δ IRV

2 Supplementary Data 2

The inhibitory effects of nisin and the GNT16 were tested against *E. coli* CIP **(Supplementary Figure 2A)** and *Enterobacter aerogenes* CECT684 **(B)**. An equal or slightly better inhibitory activity of GNT16 against both strains could be observed when comparing the growth curves of 8μM nisin and 8μM GNT16.

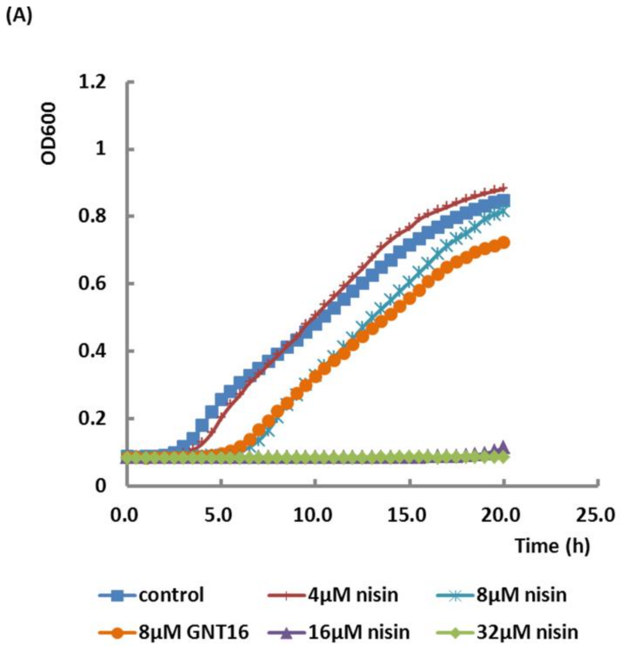

Supplementary Figure 2. Inhibitory effects of nisin and the GNT16 against *E. coli* CIP (A) and *Enterobacter aerogenes* CECT684 (B). *E. coli* CIP was grown in Luria-Bertani broth aerated by shaking (200 rpm) at 37 ℃. *Enterobacter aerogenes* CECT684 was grown in GM17 aerated by shaking (200 rpm) at 37 ℃. The growth curves were obtained with the same method as the MIC value test described in the materials and methods.

3 Supplementary Data 3

Activities of the nisin and Gram-negative tail fusions were tested either by loading TCA precipitated peptides in the plate of *L. lactis* NZ9000 (pNZnisP8H) **(Supplementary Figure 3A and B)**, or directly loading the supernatant and NisP into the plate of *L. lactis* NZ9000 (pNZ8048, pIL253) **(Supplementary Figure 3C)**, and the results indicate that all the 32 kinds of fusions made in this research showed variable activity against *L. lactis*.

(A)

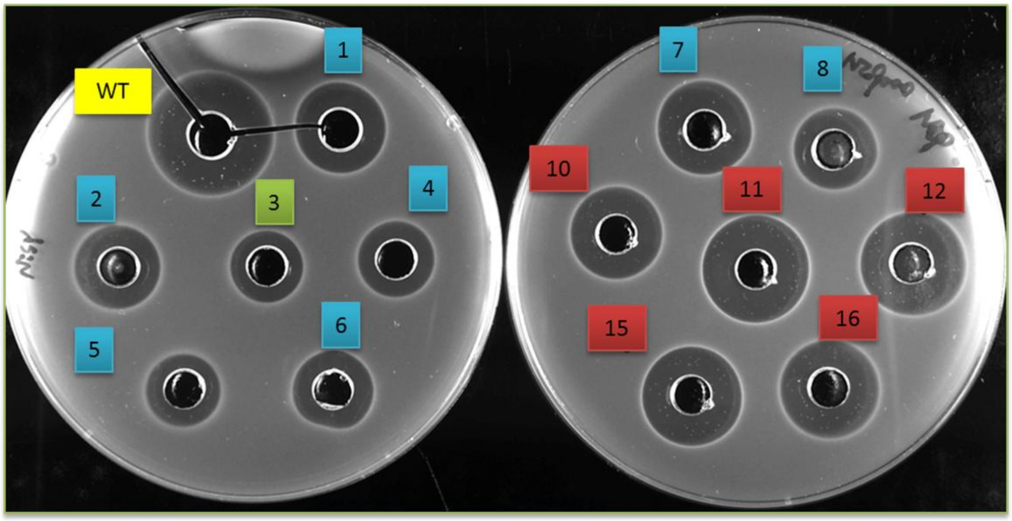
WT
1
2
3
4
5
6
7
8
10
11
12
15
16

(B)

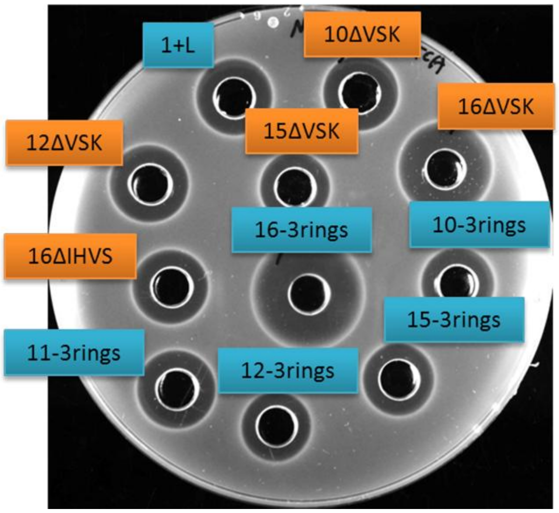
1+L
10ΔVSK
16ΔVSK
12ΔVSK
15ΔVSK
16-3rings
10-3rings
16ΔIHVS
15-3rings
11-3rings
12-3rings

(C)

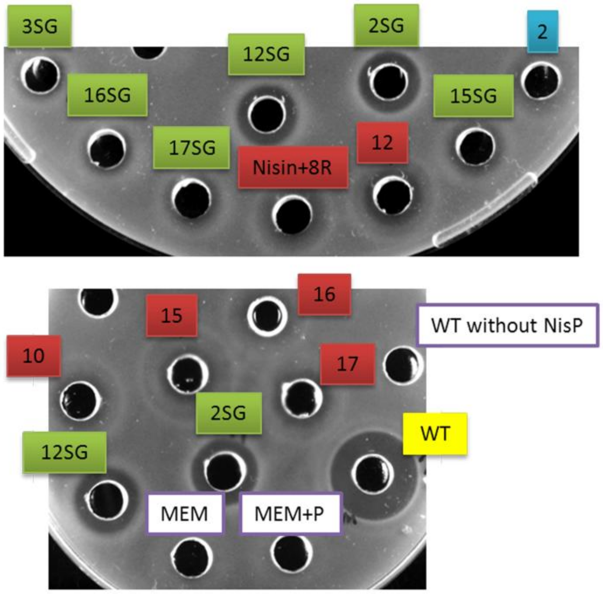

Supplementary Figure 3. Activities of the nisin and Gram-negative tail fusions against different *L. lactis* backgrounds. (A) 50µl TCA precipitated peptides (dissolved with 0.05% acetic acid) produced by NZ9000 were loaded in the plate of *L. lactis* NZ9000 (pNZnisP8H his) and 3µg/ml chloramphenicol was added to maintain the plasmid. (B) The plate is the same as (A) but 20µl TCA precipitated peptides produced by NZ9000 were loaded per well. (C) 50µl supernatant containing fusion peptides produced by PA1001 and 5µl supernatant of *L. lactis* NZ9000 (pNZnisP8H) his were loaded per well. The indicator strain is *L. lactis* NZ9000 (pNZ8048, pIL253). The controls are MEM: 50µl MEM medium; MEM+P: 50µl MEM medium with 5µl supernatant of NZ9000 (pNZnisP8H); WT without NisP: 50µl supernatant of wild type without adding NisP. The yellow label indicates wild type; blue label indicates Group 1 variants; red label indicates Group 2 variants; orange label indicates Group 3 variants; green label indicates Group 4 variants.
